# The Value of a Negative Pleural Fluid Cytology and Biopsy in Diagnosing Malignant Pulmonary Lesions

**DOI:** 10.7759/cureus.34768

**Published:** 2023-02-08

**Authors:** Henry C Egbuchiem, Nkemputaife P Onyechi, Laxmi P Sakamuri

**Affiliations:** 1 Internal Medicine, University Hospitals Geauga Medical Center, Cleveland, USA

**Keywords:** pleural fluid cytology, nodule, cytology, pleural fluid, pleural biopsy, lung cancer

## Abstract

Over 2 million patients developed lung cancer in 2018, and lung malignancy is responsible for an estimated 1.8 million deaths worldwide. Lung cancer diagnosis usually occurs after suspicious symptoms or incidental radiologic findings on chest imaging when the cancer is probably in an advanced stage. Therefore, initial evaluation, diagnosis, staging, and prompt treatment of lung cancer are required to improve pulmonary malignancies' morbidity and mortality rate. Unfortunately, the size of the tumor, the time of imaging, the quality and quantity of pleural fluid, and pleural biopsy all contribute to diagnostic difficulties in evaluating a lung lesion, leaving even the most astute clinician occasionally perplexed. We discuss a case of a female with lung cancer whose diagnosis was challenging because of a negative pleural biopsy, despite initial radiographic imaging suggesting a lung lesion.

## Introduction

Lung cancer is a leading cause of mortality worldwide [1]. In the US, in 2020, lung cancer was described as the leading cause of cancer-related deaths [2]. It is believed that over 200,000 new lung cancer cases are diagnosed yearly, with approximately over 150,000 deaths occurring annually from pulmonary cancer-related malignancy. More interestingly, despite advances in screening, diagnosis, and treatment, lung cancer's overall five-year survival rate remains low at 18% [3]. Histopathologic tissue testing and radiologic imaging are required to evaluate pulmonary lesions [3,4]. What happens when there is a discordance in the results of radiologic images and tissue histopathology? We discuss a case of a female who had a positive radiologic image for a lung lesion and a negative pleural biopsy.

## Case presentation

**Figure 1:**
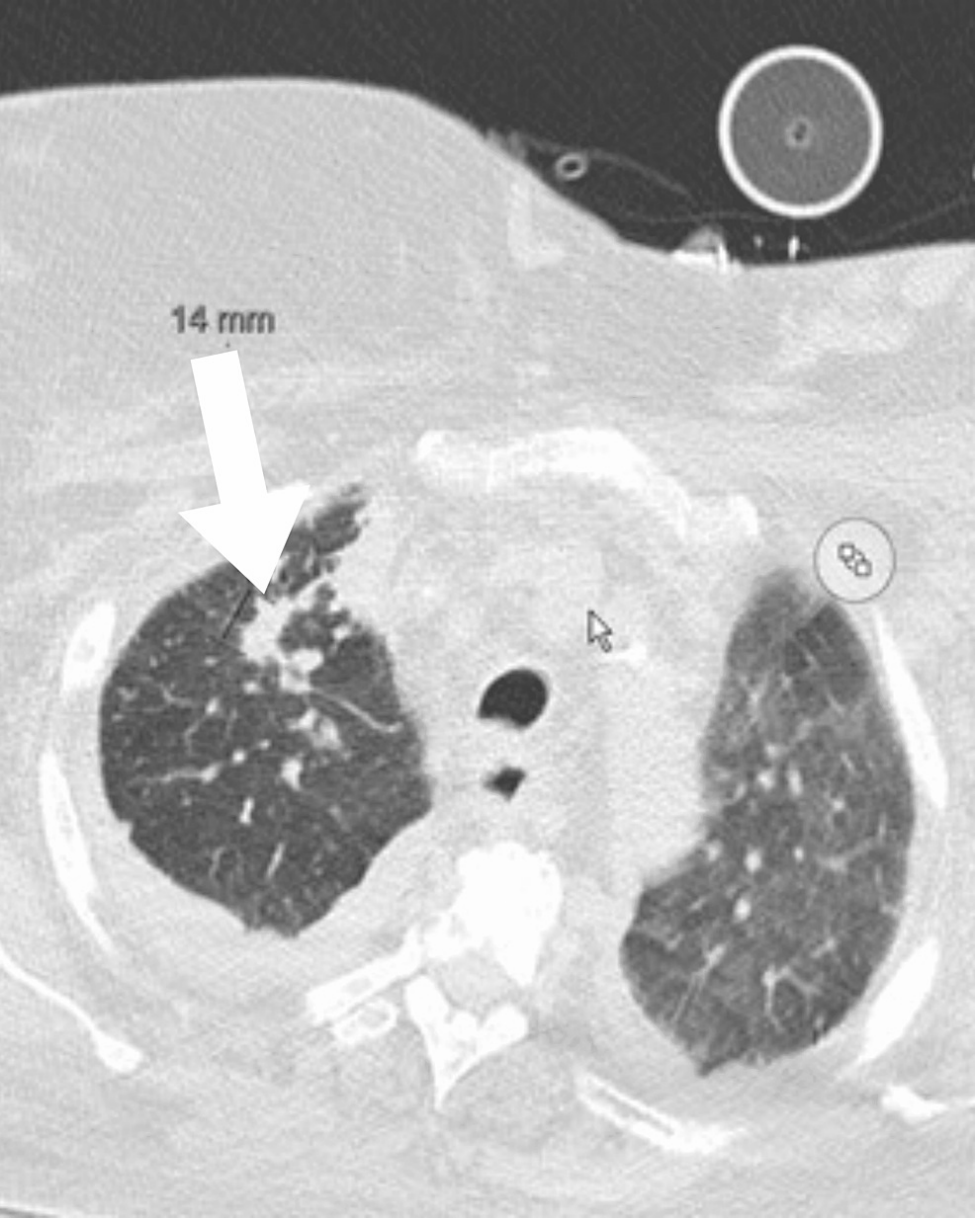
Computed tomographic image showing a 14 mm right upper lung nodule and increased bilateral hilar lymphadenopathy.

The patient was a 61-year-old, obese, African American female with a past medical history significant for Behçet's disease (on daily prednisone), superior vena cava syndrome, chronic obstructive pulmonary disease (on 3 liters home oxygen), unprovoked pulmonary embolism (on apixaban), gastrointestinal bleeding secondary to duodenal arteriovenous malformation, and history of lung nodules with mediastinal lymphadenopathy. She presented to the hospital because of intermittent chest pain and worsening shortness of breath for two days. Vital signs showed a temperature of 98° Fahrenheit, heart rate of 70 beats per minute, blood pressure of 120/85 mmHg, respiratory rate of 20 cycles per minute, and oxygen saturation of 90% on 5 liters via nasal cannula. On physical examination, she appeared to have mild respiratory distress, evidenced by the use of accessory muscles of respiration. She was alert and oriented (x3), with no motor or sensory deficit. Good air entry bilaterally with diffused rhonchi was noted, with no wheezes. She had first and second heart sounds only, with no murmurs or gallops. The rate and rhythm were regular. Soft abdomen, non-tender/distended, and no organomegaly were noted. Extremities showed no cyanosis/clubbing or edema on both lower extremities, and pedal pulses were intact. Her labs on presentation were significant for white blood cells at 20.4, hemoglobin at 6.2, hematocrit at 22.4, and platelets at 305. The metabolic panel was remarkable for elevated alkaline phosphatase at 325, negative coronavirus disease 2019 (COVID-19) test, brain natriuretic peptide at 37, and negative serial troponins. EKG showed normal sinus rhythm with no ST segment changes. CT scan showed no evidence of current embolism, with a stable right upper quadrant lung nodule with slightly increased bilateral hilar lymphadenopathy (Figure 1). There was also the presence of chronic superior vena cava occlusion with increased numbers of chest wall collateral and bony wall edema with some osseous changes. Of note, these changes were similar to her previous CT findings three months ago.

**Figure 2:**
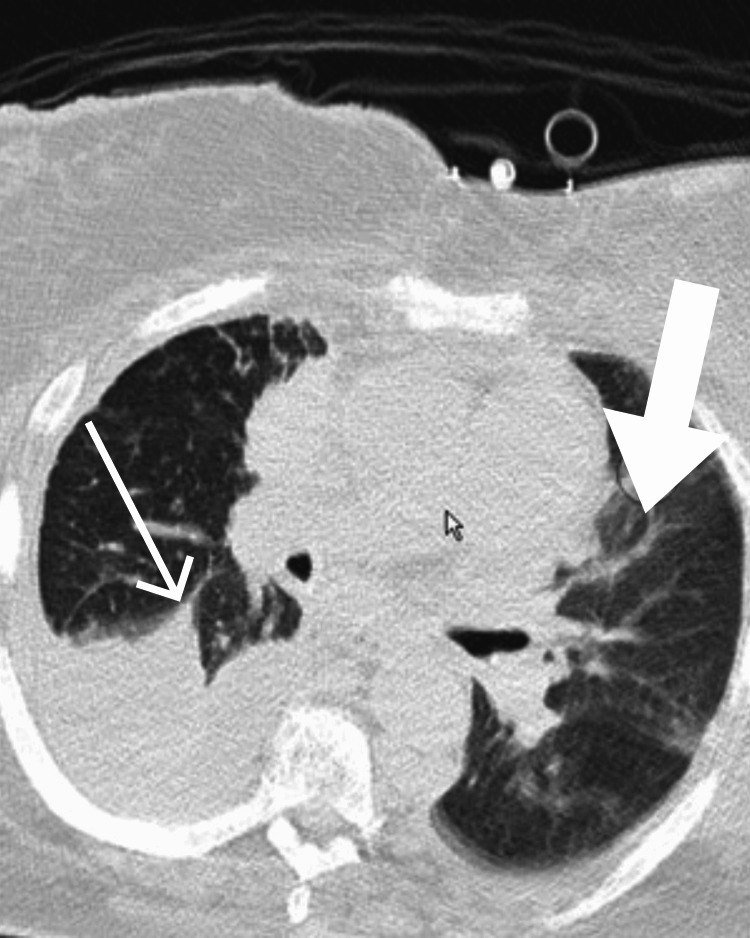
Computed tomography image showing left-loculated pleural effusion (large arrow) and right-sided pleural effusion (thin arrow).

Her social history was significant for tobacco use, with 40 pack years. A positron emission tomography (PET) scan showed multiple foci in the hip. The initial evaluation was for possible acquired pneumonia, and she received antibiotics for several days without any improvement. She then had video-assisted thoracoscopic surgery (VATS) with pleural biopsy to investigate mediastinal lymphadenopathy. The biopsy was negative for malignant cells, and she failed to extubate twice following the procedure and subsequently became dependent on ventilatory support with a tracheostomy. While in the intensive care unit, she developed anasarca that was minimally responsive to diuretics. A duplex ultrasound showed a new left upper extremity and cephalic vein deep venous thrombosis. A diagnosis of sarcoidosis was considered, given the patient's race, history of behavioral changes, bilateral hilar lymphadenopathy, osteolytic bone lesions, and a negative initial pleural biopsy ruling out malignancy. However, extensive rheumatological workup, including anti-nuclear antibodies, rheumatoid factor, creatinine kinase, antineutrophil cytoplasmic antibodies, perinuclear antineutrophil cytoplasmic antibodies, angiotensin-converting enzymes level, and autoantibodies, was unremarkable. Brain MRI showed multiple nonspecific lesions in the calvarium and skull base. Serum and urine electrophoresis both showed unremarkable results. A repeat PET CT scan was nondiagnostic as it showed decreased metabolic activity. Also, a further workup for atypical infections such as tuberculosis (TB), viral, HIV, and fungal was unremarkable. Repeat chest CT showed loculated pleural effusion in the left lung (Figure 2), and a chest tube was subsequently inserted. Pleural fluid analysis showed a transudative pattern, and pleural fluid cytology was negative for malignancy.

The possibility of lymph node biopsy was discussed and initially deferred, given her risk of bleeding. The patient underwent endobronchial ultrasound (EBUS) with bronchoalveolar lavage (BAL) and left lower paratracheal lymph node 4L biopsy. Rapid on-site evaluation (ROSE) preliminary cytology was suggestive of malignancy. The test showed adenocarcinoma of the lung with thyroid transcription factor 1 (TTF-1), napsin A, and cytokeratin (CK-7) stains positive, supporting a malignancy diagnosis.

## Discussion

There are two significant forms of lung cancer: small-cell lung cancer and non-small cell lung cancer. Non-small cell lung cancer (adenocarcinoma, squamous cell carcinoma, and large cell carcinoma) accounts for 80% of all lung cancers [4]. The definitive diagnosis of lung cancer is tissue histopathology [5]. Studies show that pleural fluid cytology after the first thoracocentesis has 60% sensitivity in diagnosing lung cancer. This value increases to 75% when repeated [6]. Hence, it is not advisable to depend solely on the absence of malignant cells in pleural cytology/biopsy for lung cancer diagnosis. In patients with imaging evidence of lesions suspicious of lung cancer, invasive tests at the location of the lesions are beneficial in staging and establishing a diagnosis [7]. As seen in our case, it is possible to get a negative result of pleural biopsy in patients with pulmonary malignancy. It is speculated that about two-thirds of lung cancers have negative pleural cytological evaluation according to Purandare et al. Their research also showed that sizeable pleural fluid is suggestive of a malignant pulmonary lesion in the absence of a positive cytologic pleural fluid. However, when a pleural fluid cytology test is negative in a suspected case of malignant pleural effusion, there are several options for further investigations, such as image-guided cutting needle biopsy, closed pleural biopsy, medical thoracoscopy, video-assisted thoracic surgery, and thoracotomy [8]. Therefore, a strong index of suspicion is required to help the clinician look for other avenues to ensure that the appropriate diagnosis is reached, irrespective of the distractors present. For example, as seen in our case presentation, we considered other possible etiology for the worsened dyspnea present in our patient. Her gender, race, and radiographic evidence of bilateral lymphadenopathy made us consider sarcoidosis as a top differential diagnosis, which was promptly discarded after further workup. Also, infectious causes like TB, fungal, viral, or atypical bugs as etiology for the dyspnea were entertained, all of which we ruled out upon further evaluation. Even though we did not initially have a histologic diagnosis supporting our suspicion of lung malignancy, we could not find any other possible explanation for the patient's shortness of breath.

Also, the approach utilized for obtaining tissue biopsy could be a factor in the test's outcome. For example, most mediastinal and central lesions are best evaluated with bronchoscopy, while more minor and peripheral lesions are best approached with a transthoracic needle for aspiration [7]. In our case presentation, the initial procedure with video-assisted thoracoscopic surgery and pleural biopsy was unremarkable and revealed no evidence of malignancy. However, when we utilized a different approach (endobronchial ultrasound with bronchoalveolar lavash) more suitable for centrally located lesions, it showed evidence of adenocarcinomatous lung malignancy.

## Conclusions

Although pleural fluid cytology/biopsy remains present in various algorithms utilized in diagnosing a lung malignancy, its low sensitivity and the preponderance of false negative results should make clinicians apprehensive. Therefore, in patients with a suspicious lung lesion and a negative pleural biopsy, further evaluation is promptly required to avoid missing or delaying the diagnosis of pulmonary malignancy.
